# Unresolved issues of immune tolerance in chronic hepatitis B

**DOI:** 10.1007/s00535-020-01665-z

**Published:** 2020-02-03

**Authors:** Hye Won Lee, Henry Lik-Yuen Chan

**Affiliations:** 1 Department of Medicine and Therapeutics, The Chinese University of Hong Kong, Shatin, Hong Kong; 2 Insitute of Digestive Disease, The Chinese University of Hong Kong, Shatin, Hong Kong; 3 Department of Internal Medicine, Institute of Gastroenterology, Yonsei University College of Medicine, Seoul, Korea

**Keywords:** Immune tolerance, Hepatitis B virus, Hepatocellular carcinoma, Antiviral therapy, Tenofovir

## Abstract

During the natural course of chronic hepatitis B virus infection, immune-tolerant phase is characterized by high viral replication, the presence of HBV e antigen (HBeAg), and normal or minimally elevated serum alanine aminotransferase. Immune-tolerant phase is usually regarded as a benign course of the disease. International guidelines recommend observation rather than treatment during immune-tolerant phase. In this article, we review unresolved issues related to the definition of true immune-tolerant phase and the benefit of antiviral treatment. Defining true immune-tolerant phase requires a careful approach and long-term follow-up. In previous studies, many patients were misclassified as being immune-tolerant phase. Noninvasive methods of assessing fibrosis are warranted for patients in the immune-tolerant phase. Yet, there has been controversy over the benefit and harm of antiviral treatment for immune-tolerant phase patients. Thus, further larger scale studies are needed to investigate the prognosis of patients in true immune-tolerant phase and their need for antiviral therapy.

## Current understanding of immune-tolerant phase

Hepatitis B virus (HBV) infection is one of the most common liver diseases worldwide. Chronic HBV infection can progress to cirrhosis or hepatocellular carcinoma (HCC). In Asia, patients with chronic hepatitis B (CHB) mostly acquire HBV infection by perinatal transmission or during infancy [1]. The majority of neonates or children exposed to HBV develop chronic infection and enter a prolonged immune-tolerant phase [1].

In perinatal acquired HBV infection, it usually comprises four phases: immune-tolerant phase, immune-clearance or active phase, inactive-carrier phase, and reactivation phase [2]. The earliest phase is characterized by very high viral replication, the presence of HBV e antigen (HBeAg), and normal or minimally elevated serum alanine aminotransferase (ALT) and/or aspartate aminotransferase (AST) [3]. This immune-tolerant phase is typically seen in patients younger than age 30 [4, 5]. The immune-clearance phase, which occurs during adolescence or adulthood, is accompanied by continuing hepatitis activity or episodic hepatitis flares. These might develop to fibrosis or cirrhosis during the HBeAg-positive phase or result in declining serum HBV DNA and HBeAg seroconversion. After HBeAg seroconversion, most patients enter the ‘inactive’ phase, which is characterized by sustained normal serum ALT and low HBV DNA [6]. However, hepatitis may relapse because of reactivation of HBV with either HBeAg seroreversion or development of precore or basal core prompter mutations. The immunopathogenesis of HBeAg-negative hepatitis is similar to that of HBeAg-positive hepatitis. Therefore, this phase is considered a variant of immune-clearance phase [7].

HBeAg is a small immunogenic secretory viral protein. HBeAg can pass through the placenta and induce clonal deletion of T cells against HBV in the fetus [8]. When the infection is acquired at birth, viral replication is usually very high, but hepatic damage is minimal. Liver biopsy typically shows no fibrosis and minimal inflammation. This can be explained by the relative lack of immune pressure on the virus [9]. Early natural history studies of immune-tolerant phase revealed a benign disease course. A study using paired liver biopsy data showed minimal progression of liver injury over 5 years among patients who remained in the immune-tolerant phase [10]. Three (6.3%, 3/48) patients had fibrosis progression and four (12.1%, 4/33) patients of F1 at baseline regressed to F0. Thus, international guidelines recommend observation rather than active treatment for patients in the immune-tolerant phase [4, 5, 11].

## Challenges on definition of immune-tolerant phase

“Defining true immune-tolerant” phase is challenging. Both natural killer (NK) and T cells are functionally impaired in the immune-tolerant and immune-active phases [12]. In one study, the proportion of NK cells in the liver was significantly higher in patients in the immune-tolerant phase compared to those in the immune-clearance phase [13]. In addition, there might be intrahepatic HBV-specific T cell activities in immune-tolerant-phase patients, even in the absence of liver inflammation [14]. A histology study in India showed that approximately 40% of HBeAg-positive patients with persistently normal ALT had necro-inflammation and fibrosis histologic fibrosis ≥ stage 2 [15]. In a retrospective cohort study in Korea, it was shown that the risk of developing hepatocellular carcinoma (HCC) was higher in untreated patients in the immune-tolerant phase than patients in the immune-active phase on antivirals (12.7% *vs*. 6.0%, *p* = 0.001) [16]. One possible reason for the poor outcome of immune-tolerant patients in previous studies is misclassification of patients in immune clearance phase as immune-tolerant. For example, in the Korean study, the mean age of the immune-tolerant patients was 38 years and 26% of immune-tolerant patients had HBV DNA 4–7 log IU/ml, which were atypical features for immune-tolerant phase [16]. In the Indian histology cohort, the lower range HBV DNA among HBeAg-positive patients with persistently normal ALT was only 2.78 log copies/ml, which was too low for immune-tolerant phase [15]. Therefore, defining immune-tolerant phase is challenging, and whether a true immune-tolerant phase exists is debatable. The 2017 European Association for the Study of the Liver (EASL) Clinical Practice Guidelines renamed this phase as HBeAg-positive chronic HBV infection instead of immune-tolerant phase to avoid confusion in terminology [11].

## Refining the definition of immune-tolerant using biomarkers

### Normal ALT level

**Fig. 1:**
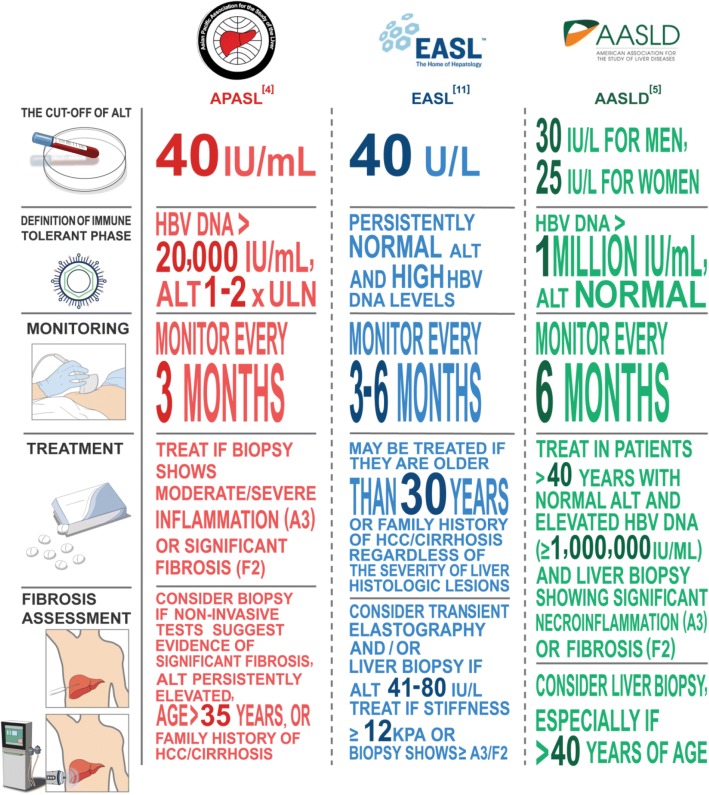
Guidelines for patients with immune tolerant chronic hepatitis B infection

Normal ALT is a key criterion to define immune-tolerant phase. The upper limit of normal (ULN) ALT in healthy subjects is 29–33 U/l for males and 19–25 U/L for females [17–19]. The American Association for the Study of Liver Diseases (AASLD) defines normal ALT as < 30 U/l for males and < 25 U/l for females [5] (Fig. 1). The Asian Pacific Association for the Study of the Liver (APASL) and EASL guidelines use the established ALT cutoff of 40 U/L [4, 11]. In the definition of normal ALT, metabolic risk factors for fatty liver disease are generally excluded. Hence a CHB patient in immune-tolerant phase can have elevated ALT if there is co-existing fatty liver disease, which has a prevalence of approximately 29.6% in Asia [20].

On the other hand, some patients with normal ALT can have liver fibrosis, as immune clearance activities might be intermittent and quiet down when ALT is checked. In a prospective study, 10% of HBeAg-positive patients with normal ALT had advanced fibrosis as assessed by transient elastography [21]. The risk for advanced liver fibrosis increases in HBeAg-positive patients older than 35 years with ALT greater than 0.5 times the ULN. Therefore, normal ALT alone in HBeAg-positive patients is insufficient to define immune-tolerant in CHB.

### HBV DNA levels

HBV DNA is expected to be very high in patients who have not experienced any immune clearance. In HBeAg-positive asymptomatic children, HBV DNA is in general > 7 log IU/ml [22] [Fig. 1]. The EASL Clinical Practice Guidelines has adopted HBV DNA > 7 log U/ml to define HBeAg-positive chronic infection [11]. AASLD guideline sets a lower bar of HBV DNA > 1 million IU/ml to define immune-tolerant phase, which has a risk of misclassifying some patients in immune-clearance phase as immune-tolerant [23]. Hence one should have a high degree of suspicion that a HBeAg-positive patient with normal ALT is not in true immune-tolerant phase but has stepped into immune clearance when HBV DNA is < 7 log IU/ml.

### HBsAg level

Quantification of hepatitis B surface antigen (HBsAg) largely reflects the concentration of covalently closed circular DNA (cccDNA) in the liver in HBeAg-positive patients [24]. HBsAg is translated from mRNA of the transcriptional active template cccDNA, which reflects the number of infected hepatocytes [25]. The mean baseline HBsAg levels differ significantly during different phases of CHB [26]. The HBsAg level is highest in immune-tolerant phase and lowest in liver cirrhosis phase: immune-tolerant (4.53 − 4.96 log_10_ U/mL), immune-clearance (4.03 − 4.37 log_10_ IU/mL), e antigen negative hepatitis (2.95 log_10_ IU/mL), low replicative (3.18 log_10_ IU/mL), and liver cirrhosis (2.69 log_10_ IU/mL) [26–28]. In one longitudinal cohort study in Hong Kong, HBsAg remained persistently high at approximately 5 log IU/mL in patients of immune-tolerant phase over a period of 8 years [29]. Thus, high HBsAg level may assist differentiation of immune-tolerant from immune-clearance in HBeAg-positive patients [30]. However, no clear HBsAg cutoff value has been identified with high sensitivity and specificity to define immune-tolerant phase.

### Liver biopsy and non-invasive assessment: impact of age

When the clinician is not certain if a HBeAg-positive patient is in the immune-tolerant phase, liver biopsy can be performed to detect evidence of necro-inflammation or liver fibrosis, which will trigger commencement of antiviral therapy (Fig. 1). According to the AASLD guideline, liver biopsy should be considered for patients with persistent borderline normal or slightly elevated ALT, in particular those 40 years or older and infected at a young age [31]. The APASL guideline states that liver biopsy should be considered for patients with abnormal transient elastography and older than age 35. The EASL guideline recommends that patients with ALT 41–80 IU/L be evaluated by transient elastography and/or liver biopsy, while antiviral therapy should be considered for patients older than age 30 even without assessment of liver fibrosis. While older age is generally agreed to be a good surrogate of immune-clearance, the cut-off age for immune-tolerant phase is unclear.

As reflected in the guidelines, liver biopsy is invasive and cannot be performed in all cases. There is an increasing need for noninvasive assessments such as transient elastography and/or serum tests. Noninvasive tests might be used to assess for the severity of fibrosis [32, 33]. Patients who have liver stiffness measurement > 8 kPa by transient elastography or APRI > 1.5 might have significant fibrosis. However, clinical application of noninvasive markers has several limitations. The diagnostic accuracy of noninvasive test is sometimes unsatisfactory. For example, AST/platelet ratio index showed moderate diagnostic performance for the assessment of fibrosis in patients with CHB [34]. Although liver stiffness measurement may have a higher diagnostic accuracy than serum noninvasive tests [35, 36], its usefulness may be reduced in obese patients [37]. In general, the specificity of liver stiffness measurement to exclude significant fibrosis in patients with normal ALT is very high [38]. Sequential use of liver stiffness measurement and serum indexes such as Forn’s index or Enhanced Liver Fibrosis test improve the accuracy to predict liver fibrosis [39, 40].

## Controversies of antiviral treatment in immune-tolerant patients

### The need to treat

There has been controversy over the need to treat patients in immune-tolerant phase. It is apparent that some patients will undergo spontaneous HBeAg seroconversion and may not need antiviral therapy. Patients with spontaneous HBeAg seroconversion before age 30 have an excellent prognosis; the 15-year cumulative incidence of cirrhosis and HCC was 3.7% and 2.1%, respectively [41]. On the other hand, older patients who remain in HBeAg-positive state may have poorer outcome, and some patients might silently move into immune clearance phase with liver injury. In one Korean study, among patients with a high viral load and normal or slightly elevated serum ALT for at least 12 months, 60% had significant fibrosis and 62% showed significant histology [42]. In Hong Kong, among HBeAg-positive patients older than age 35 with ALT greater than 0.5 × the ULN, 37% had advanced fibrosis as assessed by transient elastography [21].

The public health burden of HBV is increased by its lifelong course and the requirement for long-term follow-up [43]. There is a risk of horizontal HBV transmission from immune-tolerant patients with very high viral load. Another issue is vertical transmission of HBV by high viral load mothers. Even with hepatitis B immunoglobulin, timely birth dose and 3-dose vaccination to the newborn, approximately 5–10% of newborns will acquire HBV infection if maternal viral load is higher than 6 log IU/ml [44, 45]. Therefore, international guidelines recommend that HBeAg-positive mothers with HBV DNA > 6 log IU/mL or 200,000 IU/mL should receive oral antivirals during the last trimester, together with hepatitis B immunoglobulin and vaccination for newborns.

Most children and adults will progress from the immune-tolerant phase to the immune-active phase. There is controversy whether the risk of HCC will increase in immune-tolerant patients if they are not treated. One Korean study showed that the 10-year cumulative incidence of HCC was 12.7% among patients in the immune-tolerant phase compared to 6.0% among those in the immune-active phase, though there is concern on possible misclassification of immune-tolerant patients in this study [16]. Another Koreans study showed the contrary; the cumulative risk for HCC was similar in patients in the immune-tolerant phase and those with a virologic response groups to antivirals (1.1% and 2.7% vs. 1.0% and 2.9% at 5 and 10 years, respectively; *p* = 0.704) [46]. A study reported that suppressing HBV DNA to prevent HCC and cirrhosis is cost-effective in immune-tolerant phase patients [47]. The low cost of antiviral drugs nowadays has lowered the financial barrier for universal coverage of HBV treatment in most countries.

### Limitations of antiviral therapy

Patients in immune-tolerant phase are very difficult to treat. Patients in immune-tolerant phase respond poorly to interferon-alpha therapy [48]. In a recent study using 8 weeks of entecavir followed by combination peginterferon and entecavir therapy for 40 weeks in adults in the immune-tolerant phase, only 4% patients had HBeAg seroconversion and 0% patients had HBV DNA < 1000 IU/ml at 48 weeks after completion of peginterferon treatment [49]. A randomized controlled trial of tenofovir disoproxil fumarate (TDF) versus emtricitabine and TDF in HBeAg-positive patients with high HBV DNA (> 7 log IU/mL) and normal ALT showed that HBV DNA can be suppressed in < 60% patients and HBeAg seroconversion developed in < 5% patients in 4 years [49]. After the cessation of TDF-based treatment, all patients experienced HBV DNA relapse to > 2000 IU/mL within 4 weeks, and 50% showed an increase in ALT [50]. As a result, long-term treatment is expected if nucleot(s)ide analogue is used in immune-tolerant patients, and adherence to treatment among young patients will be a concern. Considering the minimal risk for disease progression, anticipated long-term treatment in younger patients, poor response to treatment in patients in the immune-tolerant phase, lack of data on improvement in long-term outcomes, nucleot(s)ide analogue therapy is generally not recommended for patients in immune-tolerant phase of CHB at the present moment [51].

## Summary, recommendations, directions for future research

No international consensus has been made to define true immune-tolerant phase and studies on prognosis and treatment response in true immune-tolerant phase are limited. There is no placebo-controlled study on the long-term benefit of antiviral therapy in immune-tolerant phase over observation. Given the low event rate of HCC and requirement of long-term follow-up, a randomized, controlled study to evaluate the benefit of antiviral treatment in immune-tolerant patients seems not very feasible. Most clinicians agree on the need to identify advanced fibrosis and appropriate treatment in HBeAg-positive patients with a high viral load and a normal ALT level for older patients, but the cut-off age for detailed assessment is debatable. In a true immune-tolerant phase, the risk for disease progression is minimal. Even after antiviral treatment is started, the virological response is unsatisfactory and off-treatment relapse often occurs. Thus, further studies are needed to determine the assessment and treatment strategy for immune-tolerant patients.

## Abbreviations

HBV: Hepatitis B virus
HCC: Hepatocellular carcinoma
CHB: Chronic hepatitis B
HBeAg: Hepatitis B e antigen
ALT: Alanine aminotransferase
AST: Aspartate aminotransferase
NK: Natural killer
EASL: European Association for the Study of the Liver
ULN: Upper limit of normal
AASLD: American Association for the Study of Liver Disease
APASL: Asian Pacific Association for the Study of the Liver
HBsAg: Hepatitis B surface antigen
cccDNA: Covalently closed circular DNA
qHBSAg: Quantitative HBsAg
LSM: Liver stiffness measurement
WHO: World Health Organization
TDF: Tenofovir disoproxil fumarate
